# Better beans: designer TALE-mediated discovery of common bacterial blight resistance

**DOI:** 10.1093/jxb/erae497

**Published:** 2025-01-08

**Authors:** Sujit Jung Karki

**Affiliations:** School of Agriculture and Food Science, University College Dublin, Belfield, Dublin 4, Ireland

**Keywords:** Common bacterial blight, disease resistance, OVATE family protein (OFP), transcription activator-like effectors (TALEs)

## Abstract

This article comments on:

**Gaudin C, Preveaux A, Aubineau N, Le Goff D, Jacques M-A, Chen NWG.** 2025. A dTALE approach demonstrates that induction of common bean *OVATE Family Protein 7* promotes resistance to common bacterial blight. Journal of Experimental Botany **76**, 607–620. https://doi.org/10.1093/jxb/erae433

This article comments on:

**Gaudin C, Preveaux A, Aubineau N, Le Goff D, Jacques M-A, Chen NWG.** 2025. A dTALE approach demonstrates that induction of common bean *OVATE Family Protein 7* promotes resistance to common bacterial blight. Journal of Experimental Botany **76**, 607–620. https://doi.org/10.1093/jxb/erae433

**Bacteria belonging to the genus *Xanthomonas* impact >400 plant species worldwide. They are known to employ transcription activator-like effectors (TALEs) to modulate host gene expression, leading to disease. These TALEs can be designed to target specific DNA sequences which allows them to have broad applications in genome engineering. Gaudin *et al.* (2025) showcase the use of designer TALEs (dTALEs) to identify resistance genes against common bacterial blight of bean (CBB), a significant threat to bean production worldwide. Their findings identify three genes, *PvOFP7*, *PvAP2-ERF1*, and *PvExpansinA17*, as key for disease resistance, with *PvOFP7* showing the highest differential expression, as well as significant reduction in disease symptoms and bacterial population when induced with dTALEs. This discovery opens up new perspectives on CBB resistance by linking PvOFP7-mediated defences to heat shock protein suppression and cell wall reinforcement, in addition to offering insights into the potential to use dTALEs in breeding resistant common bean varieties.**

## Deciphering *Xanthomonas* pathogenicity: role of TALEs in disease

Plant pathogenic bacteria of the genus *Xanthomonas* have a broad host range, causing diseases in >400 plant species (Jacques *et al.*, 2016; Timilsina *et al.*, 2020). These bacteria translocate key virulence proteins known as transcription activator-like effectors (TALEs) into host cells via a type III secretion system (Yang and White, 2004; Kay and Bonas, 2009). TALEs enter the nucleus and reprogram host cells by functioning as host transcription factors. They bind to specific promoter regions of target genes and induce gene expression that alters plant development and leads to disease symptoms (Kay *et al*., 2007; Boch and Bonas, 2010; Perez-Quintero and Szurek, 2019).

**Fig. 1.**
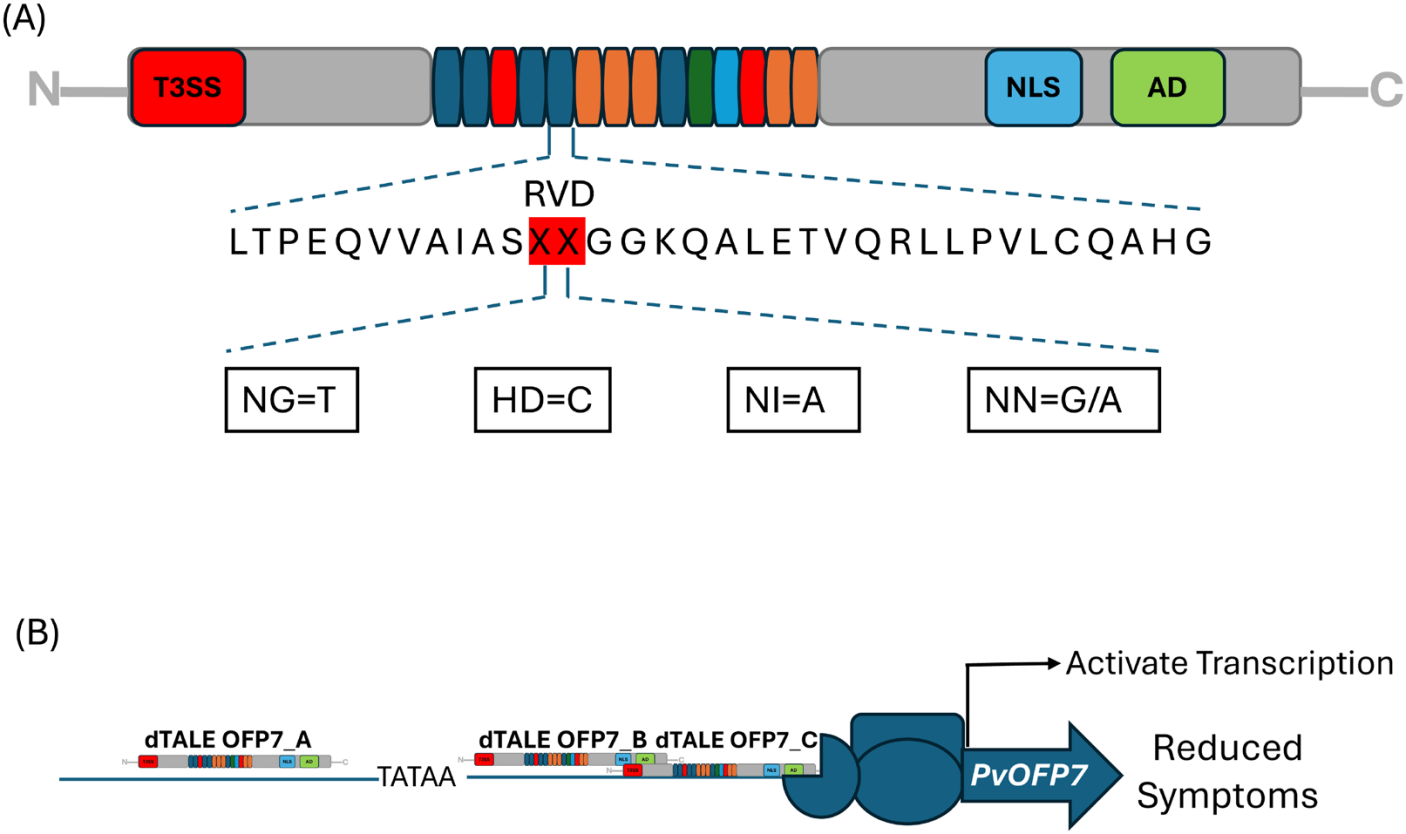
Transcription activator-like effectors (TALEs) for identification of disease-resistant genes. (A) Modular structure of TALEs with the N-terminal region containing a type III-secretion and translocation signal (red) followed by a DNA-binding module consisting of tandem repeats of 34 amino acid sequences that differ in two positions (12th and 13th) termed repeat variable residues (RVDs) shown as red XX. The DNA base specificities are represented as NG=T, HD=C,NI=A, and NN=G or A. The DNA-binding domain is flanked by a nuclear localization signal (NLS) and a transcription activation (AD) domain (Sanjana *et al*., 2012). (B) Designer TALEs (dTALE OFP7_A/B/C) were generated to modulate the transcription of the *OVATE Family Protein 7* (*PvOFP7*) gene in bean (Gaudin *et al.*, 2025).

TALEs are characterized by an N-terminal secretion signal domain for translocation, a central domain of tandem repeats for DNA binding, and a C-terminal region containing a nuclear localization signal (NLSs) and an acidic transcriptional activation domain (AD) (Fig. 1A) (Boch *et al.*, 2009; Boch and Bonas, 2010). The modular DNA-binding domain consists of 34 amino acid repeats with two variable amino acids at positions 12 and 13, known as the repeat variable diresidue (RVD), which determines DNA binding specificity (Fig. 1A). The final repeat, known as a half repeat, is conserved across 20 amino acids. This ‘TALE code’ enables the generation of custom TALE DNA-binding domains from the sequence of the RVD (Moscou and Bogdanove, 2009; Sanjana *et al*., 2012). In addition to their sequence-specific targeting as transcription activators, TALEs can be fused with other functional proteins such as nucleases, recombinases, or repressor domains which have expanded their applications in genome engineering (Christian *et al*., 2010; Li *et al.*, 2011).

Gaudin *et al*. (2025) show how artificially designed TALEs (dTALEs) can be utilized as tools to identify new resistance genes against common bacterial blight of bean (CBB). CBB, a bacterial disease caused by *Xanthomonas citri* pv. *fuscans* and *Xanthomonas phaseoli* pv. *phaseoli*, is a major threat to common bean (*Phaseolus vulgaris* L.) cultivation worldwide, with losses of up to 75% reported under severe conditions (Belete and Bastas, 2017; Chen *et al*., 2021). Although varying levels of resistance to CBB have been observed among several common bean lines, no major resistance gene to CBB has been characterized to date (Chen *et al*., 2021). Additionally, the lack of a reliable transformation protocol in common bean has hindered the study of genetic resistance in this important crop.

## Role of OVATE family protein in CBB resistance

To identify genes involved in common bean resistance, the authors used *X. phaseoli* pv. *phaseoli* complemented with dTALEs to induce the expression of 13 candidate genes in a susceptible bean variety. These candidate genes were previously identified based on their relatively higher expression in resistant compared with susceptible genotypes (Foucher *et al*., 2020). They identified *OVATE family protein 7* (*PvOFP7*), *APETALA2/ETHYLENE Responsive Factor 1* (*PvAP2-ERF1*), and *EXPANSIN A17* (*PvExpansinA17*) as having a significant impact on disease reduction when induced, compared with empty vector control. Among the three genes, *PvOFP7* showed the highest phenotypic impact. Its induction resulted in a marked decrease in disease symptoms and a reduction in bacterial populations, suggesting that *PvOFP7* plays a crucial role in resistance to CBB during early colonization (Fig. 1B).

## Heat shock protein suppression: a key to *PvOFP7*-mediated resistance against CBB

To further investigate the plant pathways involved in *PvOFP7*-mediated CBB resistance, the authors conducted a transcriptomic analysis in dTALE-induced *PvOFP7* samples. The differential gene expression analysis revealed a significant down-regulation of heat shock proteins (HSPs) and transcription factors. HSPs are well-known contributors to plant stress response and immunity (Jelenska *et al*., 2010; Fang *et al*., 2015). However, in the context of *PvOFP7*-mediated CBB resistance, the suppression of HSPs appears to be crucial. This is supported by multiple lines of evidence: HSP levels were significantly higher in compatible interactions compared with incompatible interactions with *Xanthomonas* (Garofalo *et al*., 2009), and HSPs were found to be down-regulated in CBB-resistant genotypes, while they were up-regulated in susceptible bean genotypes (Foucher *et al*., 2020). This shows that HSPs can have multifaceted roles, but further evidence is required to establish direct involvement of HSPs in CBB susceptibility.

## Cell wall reinforcement: important structural defence in plant resistance mechanisms

The importance of *OFP* genes in cell wall formation has been previously established using Arabidopsis mutants, where studies have demonstrated that *AtOFP1* and *AtOFP4* play a key role in regulating secondary cell wall development (Wang *et al.*, 2010; Liu and Douglas, 2015). Induction of *PvOFP7* triggered up-regulation of genes involved in cell wall formation and the biosynthesis of critical cell components such as cellulose, hemicellulose, pectin, and UDP-glucose (Gaudin *et al*., 2025). This suggests that reinforcing the cell wall could serve as an effective barrier against CBB colonization. Future investigations will be crucial in elucidating the precise mechanisms through which *PvOFP7*-mediated cell wall reinforcement mitigates disease severity.

## Enhancing disease resistance with dTALEs

The successful application of dTALEs to identify *PvOFP7* as a key gene in the defence response presents promising directions for breeding CBB-resistant common beans. Future research should explore naturally occurring variants of *PvOFP7* in wild bean relatives or heritage breeding lines. Variants that show high *PvOFP7* transcript levels, yet avoiding strong pleiotropic effects, could be crucial in establishing durable disease resistance. Further characterization of *PvAP2-ERF1* and *PvExpansinA17* would deepen our understanding of resistance mechanisms in common bean. Additionally, identification of key *Xanthomanas* virulence effectors and their interacting host targets could be explored to identify susceptibility genes and processes targeted by CBB. This could help design future disease control strategies using genome editing. The work by Gaudin *et al.* (2025) offers a valuable glimpse into crop improvement strategies, illustrating how dTALEs can be harnessed in breeding programmes to enhance crop resilience for the future.
